# Roles and regulation of Aquaporin-3 in maintaining the gut health: an updated review

**DOI:** 10.3389/fphys.2023.1264570

**Published:** 2023-11-28

**Authors:** Cui Zhu, Xiaoyan Nie, Qi Lu, Yinshan Bai, Zongyong Jiang

**Affiliations:** ^1^ School of Life Science and Engineering, Foshan University, Foshan, China; ^2^ Institute of Animal Science, Guangdong Academy of Agricultural Sciences, Guangzhou, China

**Keywords:** Aquaporin 3, gut health, barrier function, inflammation, oxidative stress, autophagy

## Abstract

Aquaporin-3 (AQP3) is a predominant water channel protein expressed in the intestine, and plays important roles in the gut physiology and pathophysiology due to its permeability to water, glycerol and hydrogen peroxide. In this review, we systematically summarized the current understanding of the expression of AQP3 in the intestine of different species, and focused on the potential roles of AQP3 in water transport, different types of diarrhea and constipation, intestinal inflammation, intestinal barrier function, oxidative stress, and autophagy. These updated findings have supported that AQP3 may function as an important target in maintaining gut health of human and animals.

## 1 Introduction

Aquaporins (AQPs) belong to a highly conserved group of major intrinsic proteins present in the cell membrane. Nowadays, at least 13 AQPs (AQP0 through AQP12) have been identified in the mammalians, which can be subdivided into three groups including classical aquaporins (AQP0, AQP1, AQP2, AQP4, AQP5, AQP6, and AQP8), aquaglyceroporins (AQP3, AQP7, AQP9, and AQP10) and unorthodox aquaporins (AQP11 and AQP12). Within the digestive tract, several AQPs including AQP1, AQP3, AQP4, AQP5, AQP6, AQP7, AQP8, AQP9, AQP10, and AQP11 have been demonstrated to be expressed abundantly in the stomach and intestines (Laforenza et al., 2005; Zhu et al., 2016; Cao et al., 2018; Zhu et al., 2023). Of these, AQP3 represents a predominant isoform distributed in the gastrointestinal tract which could selectively transport water, glycerol, and hydrogen peroxide (H_2_O_2_) (Yde et al., 2016). In recent decade, AQP3 has received tremendous attention due to its important roles in maintaining water transport, cell volume regulation, intestinal permeability, fluid secretion and absorption homeostasis (Zhu et al., 2016; Ikarashi et al., 2019a). Increasing data has indicated that the expression or alterations of AQP3 in the gut may be associated with several intestinal disorders, such as inflammatory bowel disease, diarrhea, intestinal barrier injury, irritable bowel syndrome, intestinal oxidative stress, and autophagy (Meli et al., 2018). Notably, previous study has indicated that AQPs including AQP3 represent important targets for dietary nutrients to regulate intestinal health (Lv et al., 2022). Thus, this review mainly summarized the updated progress concerning the role and regulation of AQP3 in the gut of human and animals, which are important for enhancing our understanding on this research field.

## 2 AQP3 expression in the intestine of different species

Nowadays, AQP3 has been demonstrated to be expressed in the intestines of many mammalian species (Table 1), including human (Hamabata et al., 2002), rats (Ramírez-Lorca et al., 1999; Zhao et al., 2016), mice (Zhang et al., 2017), pigs (Zhu et al., 2017; He et al., 2017), and desert hare (Zhang et al., 2019). For example, the AQP3 mRNA was detected in the distal ileum and colon while the localization of AQP3 protein was confirmed at the intestinal mucosal epithelium of patients with inflammatory bowel diseases by immunofluorescence confocal microscopy (Ricanek al., 2015). Previous study via human tissue microarrays also indicated that AQP3 was selectively targeted to the basolateral membrane in human intestine (Cohly et al., 2008), particularly with strong expression in the basolateral membranes of distal colon (Mobasheri et al., 2005). However, another study showed that AQP3 protein was restricted to the villus epithelial cells of human colon, with more intense in the apical than in the basolateral membranes (Silberstein et al., 1999). *In vitro* study also confirmed the presences of AQP3 mRNA and protein (Itoh et al., 2003) as well as the immunocytochemical localization of AQP3 in HT-29 human colon epithelial cell line (Li et al., 2015).

**TABLE 1:** The expression of AQP3 in the intestines of different species.

Species	Small intestine			Large intestine	Cell types	References
Human	—	—	Ileum	Colon	HT-29, Caco-2	Ricanek al. (2015); Mobasheri et al. (2005); Silberstein et al. (1999); Itoh et al. 2003; Li et al. (2015)
Rat	—	Jejunum	Ileum	Colon	—	Ramírez-Lorca et al., 1999
						Laforenza et al., 2005
						Zhao et al. (2016)
Mouse	—	Jejunum		Colon	—	Zhang et al. (2017)
Pig	Duodenum	Jejunum	Ileum	—	IPEC-J2, IPEC-1	He et al. (2017); Zhu et al. (2017a); Wu et al., 2020; Krone et al., 2019
Desert hare	Duodenum	Jejunum	Ileum	Colon	—	Zhang et al. (2019)
Broiler	—	Jejunum	—	—	—	Alhotan et al. (2021)

“—” indicates no related publications found.

In rats, the AQP3 mRNA was found in the middle portion of small intestine, ileum, proximal colon and distal colon segments, while AQP3 protein was detected abundantly in the basolateral plasma membrane of colon surface columnar epithelial cells (Koyama et al., 1999; Ramírez-Lorca et al., 1999; Yde et al., 2016; Zhao et al., 2016). Another study has also indicated that the expression of AQP3, AQP4, and AQP8 in superficial colonocytes was higher than the lower crypt cells of rat proximal colon (Laforenza et al., 2005). In fact, the immunofluorescence analysis clearly showed that AQP3 expressed at both the apical and basal sides of mucosal epithelial cells in rat colon (Ikarashi et al., 2016), confirming that AQP3 was the dominant AQP subtype in the luminal side of rat colon mucosa compared to AQP4 and AQP8 (Ikarashi et al., 2011a). Moreover, the AQP3 mRNA expression was downregulated in jejunum tissue samples of rats treated by heat stroke, while AQP3 localization was present from the lamina propria to the crypt following heat stroke (Wang et al., 2015). However, the expression of AQP3 in the rat residual ileum and colon was upregulated during the adaptation after an 80% small bowel resection on postoperative day 1 and 7 (Tsujikawa et al., 2003).

In mice, AQP3 was mainly localized to the jejunum villi epithelial cells of mice rather than the duodenum or ileum (Zhang et al., 2017). Moreover, AQP3 has also been found to be localized at the apical side of mice colonic epithelial cells (Wang et al., 2017). Notably, the AQP3 localization shifted from its normal location along cell membranes to the cell cytoplasm during bacterial infection in C57BL/6 mice (Guttman et al., 2007).

In pig, the AQP3 was widely distributed in different sections of small intestine of piglets, with more abundant expression in the duodenum than jejunum and ileum (He et al., 2017). However, another study showed that AQP3, AQP7, and AQP10 expression was greatest in the jejunum of growing pigs (Krone et al., 2019). We previously also found that AQP3 was expressed in the duodenum, jejunum, and ileum of weaned piglets (Zhu et al., 2017).

Interestingly, AQP3 was found to be located in the small intestinal villus epithelium and colonic epithelium of a desert hare, *Lepus yarkandensis* (Zhang et al., 2019). To date, though limited research was conducted on poultry regarding the AQPs distribution and expression, the transcripts of AQP1, AQP3, and AQP9 were evident in the jejunum of broilers (Ross 308) (Alhotan et al., 2021).

## 3 The water transport in the gastrointestinal tract and potential involvement of AQP3

The gastrointestinal epithelium plays a major role in the control of fluid and ion balance (Turvill and Farthing, 1999; Laforenza, 2012). For the water transport and fluid absorption in gut epithelium, it often involves both the paracellular route and the transcellular route (Laforenza, 2012). The paracellular route is mainly mediated by the space between cell tight junctions and is quite limited in small intestine with leaky epithelium (Masyuk et al., 2002). Increasing evidence has indicated that there are several potential mechanisms to mediate the transcellular route of water transport in gut epithelium, which include 1) the passive diffusion through the phospholipid bilayer (Benga, 2009), 2) the cotransporter with ions and nutrients (Wright and Loo, 2000; Zeuthen, 2010), and 3) the osmotically-driven transmembrane water movements through AQPs (Ma and Verkman, 1999; Benga, 2009; Mollajew et al., 2010; Laforenza, 2012; Ye et al., 2023). Thus, the expression of AQP3 in gastrointestinal tract of different species may suggest an involvement in water transport through a transcellular route. Numerous studies have suggested the important role of AQP3 in water transport and fluid absorption of gut, and the alterations of AQP3 expression in the gut may be associated with intestinal orders such as diarrhea or constipation (Camilleri et al., 2019; Yde et al., 2021). For example, the inhibition of AQP3 function in the colon using HgCl_2_ or CuSO_4_ would contribute to increased fecal water contents and develop severe diarrhea in rats (Ikarashi et al., 2012a). Similar results have showed that mercury exposure would induce significant reductions of protein expression of AQP3 in the stomach, jejunum and colon of rats (Bottino et al., 2016). Another study also reported that both AQP3 mRNA and protein expression was decreased in patients with or without bile acid malaabsorption (Camilleri et al., 2019). Based on these findings, the altered expression of AQP3 in the intestine might cause disorder of water transfer and fluid metabolism, which indicated the potential role of AQP3 in influencing the fluid homeostasis and gut health (Koyama et al., 1999; Zhu et al., 2017). However, the precise assessment of the functional role of AQP3 under physiological and pathophysiological conditions still requires to be fully defined in future studies (Yde et al., 2016).

## 4 Role and regulation of AQP3 expression in diarrhea

### 4.1 Irritable bowel syndrome-diarrhea

Irritable bowel syndrome (IBS) is a chronic functional gastrointestinal disorder that affects 9%–23% of the population across the world (Saha, 2014; Radziszewska et al., 2023). Abdominal pain associated with defecation disorder and fluid balance disturbance, was the predominant bowel dysfunction in patients with IBS-D (Camilleri, 2015). Previous study has showed that the AQP3 expression at both mRNA and protein levels was decreased in rectosigmoid mucosal biopsies of patients with IBS-D (Camilleri et al., 2019). Further study showed that the levels of long noncoding RNA H19, AQP1, and AQP3 were all reduced in the colonic mucosa of IBS-D patients, and the reduced AQP1 and AQP3 protein expression in Caco-2 cells was positively correlated with the level of long noncoding RNA H19 (Chao et al., 2020). However, previous study in rats showed that atractylodes oils could alleviate the predominant IBS-D and reduce intestinal inflammation by partially increasing the expression of AQP3 in the colon (Xie et al., 2021).

### 4.2 Enterotoxigenic *Escherichia coli* (ETEC)-induced diarrhea and weaning

ETEC is well-recognized as a major cause of diarrhea in neonates, children and travelers in lower-income countries (Zhang et al., 2022), and ETEC-induced diarrhea also represents an important problem in swine production especially during the weaning period (Kim et al., 2022). Increasing data has indicated the important role of AQP3 in ETEC-induced diarrhea. For example, we previously demonstrated that ETEC K88 induced significant changes of ion transporter and water channel proteins, with AQP3 expression in the intestine of weaned piglets significantly downregulated by ETEC challenge (Zhu et al., 2017). Similarly, Zhang et al. (2017) also found that the AQP3 expression was gradually decreased after ETEC administration for 1, 3, 5, or 7 days compared to the unchanged control group. Moreover, the protein expression of AQP3 in colonic mucosa of piglets was significantly decreased when challenged with either recombinant *Escherichia coli* expressing heat-stable enterotoxin or ETEC K88 (Lv et al., 2018). Besides, both AQP2 and AQP3 localizations would be altered from cell membranes to cell cytoplasm after bacterial infection depending on the bacterial type III effector proteins EspF and EspG (Guttman et al., 2007). This was true since the altered localizations of AQPs may represent an important contributing factor to diarrhea during bacterial infection (Guttman et al., 2007). Increasing data has suggested that AQP3 has an important role in the reduction of ETEC-induced diarrhea for potential dietary interventions. For example, previous study has showed that *Lactobacillus plantarum* strains FCQNA30M6 and CCFM1143 alleviated ETEC-induced diarrhea and increased the serum concentration of AQP3 in mice (Yue et al., 2020). Another study also found that immunohistochemistry scores of AQP3 in colon cancer patients were positively correlated with serum AQP3 concentrations (Hong et al., 2020). A recent study has also shown that dietary berberine supplementation significantly upregulated the mRNA expression of aquaporins (AQP1, AQP3, AQP4, AQP7, and AQP10) and Na^+^/H^+^ exchanger 3 in ETEC K88-challenged piglets (Zhu et al., 2023). However, other ETEC strains (SEC470, SEC 298; SEC 817 or C197) had little effect on the protein abundance of AQP3 in the jejunum of mice at 24 h post ETEC infection (Wu and Su, 2018).

Zinc oxide (ZnO) has been well demonstrated to alleviate the postweaning diarrhea of piglets. Peng et al. (2020) found that dietary supplementation with either pharmacologic dose (3,000 mg/kg) of conventional ZnO or porous ZnO at 750 or 1,500 mg/kg could reduce the mRNA expression of AQP3 in the jejunal mucosa, and alleviate the incidence of diarrhea of weaned piglets. In contrast to ZnO, dietary supplementation with an amino acid blend (glutamate: glutamine: glycine: arginine: N-acetylcysteine = 5: 2: 2: 1: 0.5) could significantly upregulate the mRNA expression of AQP3, AQP8, and AQP10 as well as the protein expression of AQP3 and AQP4 in the jejunum, and reduce the incidence of diarrhea with an enhanced intestinal function and growth performance in weaned piglets (Yi et al., 2018). The discrepancy of these results may be related to the differences in experimental design, sampling time, management, and the potential interactions of AQP3 with other AQPs. Thus, further investigations concerning the possible mechanism of different nutritional interventions on diarrhea through AQP3 regulation in weaned piglets.

In summary, most of these findings indicated that the inhibition or reduced AQP3 expression was closely associated with regulation of water transport and absorption and fluid homeostasis in the gastrointestinal tract. AQP3 may represent an important target for the nutritional regulation of neonates and piglets during the critical weaning period when they are vulnerable to ETEC infection.

### 4.3 Porcine epidemic diarrhea and rotavirus diarrhea

Porcine epidemic diarrhea virus (PEDV) causes a highly contagious intestinal disease in neonatal and weaned piglets with the symptoms of acute diarrhea, vomiting, and severe dehydration porcine epidemic diarrhea (Wang et al., 2022). It has been demonstrated that AQP3 expression was significantly decreased in the small intestine of PEDV-infected piglets as well as in Sp1-inhibited porcine intestinal epithelial cells (IPEC-J2), and showed a negative correlation with the increased methylation levels of mC-20 site of CpG1 and mC-10 site of CpG2 in the AQP3 promoter region (Wu et al., 2020). In line with this, downregulation of AQP3 by shRNA silencing in IPEC-J2 cells increased the genome copies and viral titers of PEDV, which proved that AQP3 could inhibit the PEDV infection in IPEC-J2 cells (Wang et al., 2022). Moreover, in a mouse model of rotavirus diarrhea, the AQP3 expression in the colon was significantly upregulated, while AQP1, AQP4, while AQP8 expression was downregulated by rotavirus challenge (Cao et al., 2014).

### 4.4 Other types of diarrhea

#### 4.4.1 Spleen deficiency-induced diarrhea

In traditional Chinese medicine, the spleen-deficiency diarrhea is regarded as a prevalent gastrointestinal condition with diarrhea as the primary symptom (Fan et al., 2023). The AQP3, AQP4, and AQP8 expression of colon was significantly decreased in rats with spleen-deficiency diarrhea, while their expression was significantly increased by administration with Pingwei San or Shenling Baizhu San (Fan et al., 2023). Similarly, treatment with a high dose of *Atractylodis Rhizoma* extract for consistent 10 days help prevent the spleen deficiency-induced diarrhea and reduce the pathological changes in colon tissue of mice, which might be associated with the enhanced expressions of both AQP3 and AQP8 in the colon (Shi et al., 2019). Moreover, as reported in a recent study, the treatment with ethanol extract of deep-fried *Atractylodes lancea Rhizome* significantly improved the symptoms of mice with spleen-deficiency diarrhea by partially promoting the expression of AQP3, AQP4, and AQP8 and tight junction markers (Liu et al., 2023). Another study showed that oral administration of both low dose and high dose of Ershen pill extract could prevented body weight loss and inhibited diarrhea after 2-week treatment (Pan et al., 2019). The anti-diarrhea mechanism of Ershen pill extract might be associated with the regulation of AQP3, since high dose of Ershen pill extract significantly improved the AQP3 positive staining intensity as well as the AQP3 protein synthesis in the colon tissue of rats with spleen deficiency-induced diarrhea (Pan et al., 2019).

#### 4.4.2 Antibiotic-associated diarrhea

Antibiotic-related diarrhea is known as a common side-effect during antibiotic treatment inducing dysbacteriosis of gut microbiota, with *Clostridium difficile* infection being the most common causative agent of the disease (Li et al., 2023). The incidence of antibiotic-related diarrhea remains relatively high especially in hospitalized children and older adults (Kaya et al., 2023; Zhao et al., 2023). The effectiveness of probiotic strains in the prevention and treatment of antibiotic-associated diarrhea has been well-summarized in recent systematic literature reviews (Doar and Samuthiram, 2023; Yang et al., 2023). However, few studies could be found concerning probiotic regulation on antibiotic-associated diarrhea by targeting AQPs. There was such one study showing that *Bacteroides fragilis* strain ZY-312 could alleviate the antibiotic-associated diarrhea in rats by enhancing the colonic AQP3 and AQP8 expression and modulating intestinal defenses (Zhang et al., 2018).

#### 4.4.3 Bile acid-induced diarrhea

Bile acid-induced diarrhea is usually caused by an excess of bile acids in the colon due to bile acid malabsorption (Yde et al., 2016), which is very common in patients with inflammatory diseases, such as ileal resection, Crohn’s disease, and diarrhea-predominant irritable bowel syndrome (Duan et al., 2019). An earlier study indicated that the mechanism of bile acid-induced diarrhea might be associated with an increase in mucosal permeability to ion and water and mucosal damage (Argenzio and Whipp, 1983). These authors found that the ion transport and net water absorption in the pig colon were abolished by deoxycholic acid, accompanied with an increased mucosal permeability (Argenzio and Whipp, 1983). Previous study in rats fed with cholic acid also revealed that a reduced AQP3 protein levels in colonic epithelia and increased fecal water contents, indicating its involvement in the pathophysiology of bile acid-induced diarrhea (Yde et al., 2016).

#### 4.4.4 Irinotecan-induced diarrhea

Irinotecan is a broad-spectrum cytotoxic anticancer agent with a series of toxic side-effects (Yue et al., 2021). A recent systematic review and meta-analysis study has demonstrated that Chinese herbal medicine represented an effective complementary and alternative prevention and therapy for irinotecan induced diarrhea (Lin et al., 2023). Indeed, the herbal medicines and their derived phytocompounds might be effective complementary treatments for irinotecan induced diarrhea compared to the generally recommended treatments with high-dose loperamide and octreotide of controversial effects (Tang et al., 2014). In contrast, another study suggested a lack of benefit of administered probiotic formula containing *Bifidobacterium* and *Lactobacillus rhamnosus* for the prevention of irinotecan-induced diarrhea in colorectal cancer patients (Mego et al., 2023). Interestingly, the incidence of severe irinotecan-induced diarrhea in patients with advanced colorectal cancer has been significantly reduced by the application of activated charcoal (Michael et al., 2004). By inducing severe diarrhea, irinotecan has been demonstrated to cause a significant decrease of AQP3 expression in the colon of rats, while anti-inflammatory drugs celecoxib could prevent irinotecan-induced delayed diarrhea by suppressing the reduction of AQP3 expression (Kon et al., 2018a).

#### 4.4.5 Castor oil-induced diarrhea

Castor oil is mainly used to treat elderly or patients with long-term constipation due to its laxative property (Arslan and Eşer, 2011). However, excess intake of castor oil might induce diarrhea characterized by disordered water and ion balance. Previous study has found that the fruit extracts of Capsicum annum L. abundant in Pakistan could significantly inhibit the number of defecations in castor oil-induced diarrhea by increasing the urinary excretion of water and electrolytes (Mazhar et al., 2022). Similarly, Camelina sativa oil treatment could relieve the castor oil-induced diarrhea by modulating gut microbiota composition and the production of short-chain fatty acids and reducing serum proinflammatory indices in mice (Zhu et al., 2022). Nevertheless, other investigators showed that castor oil increased the mRNA expression of AQP3, but the anti-diarrhea effect of Chinese herb medicine FengLiao was associated with a decreased AQP3 expression in the jejunum of mice with castor oil-induced diarrhea (Chen et al., 2020).

#### 4.4.6 Senna-induced diarrhea

Senna and its main component sennosides are recognized as effective laxative drugs for treating intestinal constipation (Cao et al., 2018). However, clinical side-effects of diarrhea and toxicity on the kidneys and livers should be cautioned (Cao et al., 2018). These authors demonstrated that high doses of both senna extract and sennosides treatments resulted in a significant diarrhea grade, with colonic AQP3 mRNA expression upregulated in all diarrhea groups (Cao et al., 2018). In a mice model of senna-induced diarrhea, the transdermal administration of Renzhu ointment has been shown to significantly reduce the frequency of loose stools, diarrhea rate and index, and fecal moisture content (Zhong et al., 2023). Moreover, the BALB/c mice developed acute diarrhea via senna extract solution in accompany with a decreased expression of AQP3 in the jejunum, while interventions of *Malus pumila* leaf flavonoids at either 25 mg/kg or 50 mg/kg by gavage displayed antidiarrheal effects by increasing AQP3 expression (Yi et al., 2020).

### 4.5 Cyclic adenosine monophosphate regulation of AQP3 expression in the development of diarrhea

**FIGURE 1:**
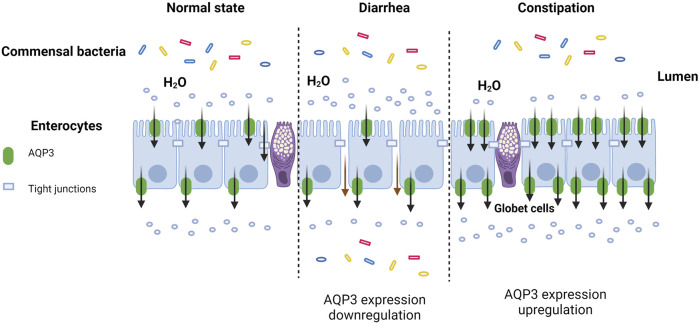
AQP3 as a new therapeutic target in diarrhea and constipation.

cAMP is known as the second messenger in the body. Accumulating evidence indicated that AQP3 expression could be regulated by cAMP pathway mainly through protein kinase A (PKA) and its downstream effectors such as cAMP-responsive element binding protein (CREB). This might be due to the fact that the 5′-flanking region of AQP3 gene harbors promoter activity and two binding sites of the transcription factor CERB (Yde et al., 2016). Previous study has found that cholera toxin treatment negatively regulated osmotic water permeability of AQP3 and AQP4 by increasing intracellular cAMP concentration (Hamabata et al., 2002). Moreover, patchouli oil could significantly decrease the expression of AQP3, AQP7, and AQP11 in the small intestine of rats via the vasoactive intestinal peptide (VIP)-cAMP-PKA signaling pathway, thereby restoring the intestinal water absorption for alleviating 5-fluorouracil-induced diarrhea (Gan et al., 2020). Notably, the antidiarrhoeal effect of Rhubarb tannins extract might be associated with its reduction in the expression of AQP2 and AQP3 in apical and lateral mucosal epithelial cells in the colon of diarrhea mice as well as in HT-29 cells induced by MgSO_4_ via downregulating PKA/CREB signal pathway (Liu et al., 2014). Another study also found that β-patchoulene ameliorated water transport and alleviated diarrhea by inhibiting AQP3 function via the cAMP/PKA/CREB signaling pathway in intestinal mucositis-induced rats (Wu et al., 2021). These results indicated that AQP3 may act as a new therapeutic target (Figure 1, created with BioRender.com) in diarrhea (Wu et al., 2021), and the activation of cAMP/PKA/p-CREB pathway was involved in regulating AQP3 expression in colonic epithelial cells.

## 5 Role of AQP3 in constipation and regulation of laxatives on its expressionin the colon

Constipation is characterized as a functional bowel disorder with a difficult, irregular, or deficient defecation (Longstreth et al., 2006). Normally, the colon would absorb approximately 1.5–2.0 L of water daily via both transcellular and paracellular routes (Laforenza, 2012). Increasing data has indicated that AQP3 in the colon was closely related to fecal characteristics and was involved in the onset of constipation probably by promoting water absorption from the luminal side to the vascular side (Table 2). For example, AQP3 mRNA expression in the colon was upregulated with increasing serotonin (namely, 5-hydroxytrypamine, 5-HT) secretion in constipation-rats induced by morphine (Kon et al., 2015). Indeed, both AQP3 and AQP8 were upregulated in the colonic biopsies of patients with functional constipation (Lin et al., 2023). Moreover, the combination of HgCl_2_ and morphine could improve the morphine-induced constipation in mice (Kon et al., 2015). In a mouse model of constipation established by diphenoxylate, the expression of AQP3 in colonic mucosa was also found to be significantly upregulated (Sun et al., 2018). Similar pattern of increased AQP3 expression was found while using diphenoxylate hydrochloride to induce constipation of rats (Cong et al., 2019). Thus, most of these findings supported the notion that increased AQP3 might be an important characteristic of most functional constipations (Yuan et al., 2008). Notably, the use of diphenoxylate at much higher dosage for a long time would result in an opposite result, with colonic AQP3 expression downregulated in these constipation models (Zhan et al., 2020; Tan et al., 2021). Thus, these studies suggest that AQP3 functions as a new promising target for the development of laxatives (Ikarashi et al., 2012a), and may act as a new therapeutic target in alleviating the constipation (Ikarashi et al., 2016) (as summarized in Table 3).

**TABLE 2:** The expression of AQP3 in different constipation models.

Species	Constipation-induced drugs	Dosage	AQP3 expression	Level	References
Rat	Morphine	20 mg/kg	Colon↑	mRNA, protein	Kon et al. (2015)
Rat	Loperamide	5 mg/kg for 12 days	Colon↑	Protein	Na et al. (2022)
Mouse	Loperamide	80 mg/kg in 0.5% carboxymethy cellulose sodium	Colon↑	Protein	Wang et al. (2023b)
Mouse	Loperamide	3 mg/kg weight for 5 days	Colon↓	mRNA, protein	Yin et al. (2018)
Rat	Diphenoxylate hydrochloride	3 mg/kg for 14 days	Colon↑	Protein	Cong et al. (2019)
Mouse	Diphenoxylate	0.1 mL/10 g	Colon↑	Protein	Sun et al. (2018)
Mouse	Diphenoxylate	20 mg/kg for 4 weeks	Colon↓	mRNA	Tan et al. (2021)
Rat	Diphenoxylate	10 mL/kg for 14 days	Colon↓	Protein	Zhan et al. (2020)
Mouse	Diphenoxylate	10 mg/kg for 5 days	Colon↑	mRNA, protein	Hu et al. (2019)

**TABLE 3:** Regulation of laxatives on the expression of AQP3 in the constipation models of rodents.

Category	Laxative	Species	Dosage	Method	AQP3 expression	Constipation alleviation effect	References
Osmotic laxatives	MgSO_4_	Rat	2 g/kg	Oral administration	Colon↑	Increased fecal water content	Zhu et al. (2019a)
	Lactulose	Mouse	3 g/kg lactulose	Gavage treatment	Colon↑	Increased fecal number, fecal weight and fecal water content	Tan et al. (2021)
	Fructose	Rat	Not mentioned	Feed	Unchanged	Fructose did not improve constipation	Cong et al. (2019)
Stimulant laxatives	Bisacodyl	Rat	20 mg/kg body weight	Oral administration	Colon↓	Increased water content after 5 h adminstration	Ikarashi et al. (2011b)
	Bisacodyl	Rat	0.25 mg/kg	Oral administration	Colon↓	Increased fecal number and weights	Na et al. (2022)
	Emodin	Mouse	1, 2 and 3 g/kg/day	Oral administration	Colon↑	Increased the evacuation index of defecation and fecal water content	Zheng et al. (2014)
	Daiokanzoto	Rat	1.5 g/kg	Oral administration	Colon↓	Increased total weight of feces and fecal water content	Kon et al. (2018a)
	Rhubarb extract	Rat	1 g/kg body weight	Oral administration	Colon↓	Increased fecal water content	Kon et al. (2014)
	Sennoside A	Rat	50 mg/kg body weight	Oral administration	Colon↓	Increased fecal water content	Kon et al. (2014)
	Senna extract	Rat	1725 or 3,450 mg/kg	Gavage treatment	Colon↓	Not determined	Cao et al. (2018)
Serotonergic agents	Fluoxetine	Rat	10 mg/kg	Intraperitoneal injection	Colon↓	Increased number of fecal pellets and weight of pellets	Kon et al. (2015)
	*Lactococcus lactis* subsp. lactis HFY14	Mouse	1×10^8^ or 1 × 10^9^ CFU/kg	Gavage treatment	Colon↑	High dose of HFY14 increased fecal number, fecal weight and fecal water content	Tan et al. (2021)
	Mixture of *B. breve* DM8310, *L. acidophilus* DM8302, and *L. casei* DM8121	Rat	1 × 10^9^ CFU/kg	Intragastric administration	Colon↑	Increased fecal water content and improved small intestine transit	Deng et al. (2018)
	Dietary fiber	Rat	5%, 10%, and 15% high specific volume polysaccharide	Feed	Colon ↓	Increased fecal relative moisture content and reduced fecal hardness at medium or high doses	Cong et al. (2019)
	Dietary fiber	Rat	5% partially hydrolyzed guar gum	Feed	Colon ↓	Increased fecal water content	Kon et al., 2022
Lubricating agents	Rhein	Mouse	0.1 mL/10 g body weight	Gavage treatment	Colon↓	Reduced first defecation and increased red stools number	Sun et al. (2018)
Others	Citric acid-enriched extract of ripe *Prunus mume*	Rat	100 or 200 mg/kg	Oral administration	Colon↓	Increased number of feces, fecal weights and fecal moisture	Na et al. (2022)
	Resin glycoside fraction	Rat	62.5 or 125 mg/kg	Oral administration	Colon↓	Watery stool was obvious after 6 h adminstration	Zhu et al. (2019b)
	Mulberry	Mouse	62.5, 125, and 250 mg/mL mulberry powder	Oral administration	Colon ↓	Increased fecal water content and shorten the first red fecal defecation time	Hu et al. (2019)
	Naringenin	Mouse	300 mg/kg body weight, once a day for 5 days	Intragastric administration	Colon↑	Increased fecal number, fecal weight, fecal water content, and gastrointestinal transit	Yin et al. (2018)

### 5.1 Effect of osmotic laxatives for constipation on the expression of AQP3

Magnesium sulfate (MgSO_4_) represents as an important osmotic laxative, could increase the protein expression of AQP3 in the colon of rats (Ikarashi et al., 2011a; Zhu et al., 2019). Whereas the concomitant use of MgSO_4_ and bisacodyl induced a lower content of fecal water content than in the MgSO_4_ group, but increased the expression of AQP3 in the colon without enhancing the laxative effect (Ikarashi et al., 2012b).

Lactulose is another commonly used osmotic laxative in treating constipation. Previous study has shown that lactulose treatment at 3 g/kg for 4 weeks effectively alleviated the constipation damage in the colon of mice by upregulating the AQP3 expression through regulating the cAMP-PKA pathway (Tan et al., 2021). Similar results of AQP3 changes have been found when administrating lactulose (500 mg/kg) daily for 14 days in loperamide-induced functional constipation mice (Wang et al., 2023).

Fructose has also been shown to resolve symptoms in patients with chronic functional constipation (Mehta and Beg, 2018). This might be because the increased ingestion of fructose would increase fructose malabsorption with symptoms of insufficient absorption and subsequent bacterial fermentation of fructose, which resulted in a state of fructose intolerance to increase the intestinal motility for inducing looser stools (Mehta and Beg, 2018). Indeed, another study confirmed that the fructose intolerance and malabsorption was quite common in functional gastrointestinal disorders (Wilder-Smith et al., 2013). However, fructose treatment did not significantly affect the AQP3 protein expression in the colon of rats compared to those in the constipation model group (Cong et al., 2019).

### 5.2 Effect of stimulant laxatives for constipation on the expression of AQP3

AQPs represent important targets for traditional Chinese medicine in the treatment of digestive disorder including constipation (Huang et al., 2022). A recent study showed that Xuanhuang Runtong tablets could reverse the disorder of AQP3 expression and thus relieve slow transit constipation in mice (Liang et al., 2023). Moreover, the laxative effect of Daiokanzoto has been confirmed when used as a long-term treatment for chronic constipation probably by maintaining a persistent decrease of AQP3 expression and gut microbiota homeostasis (Kon et al., 2018b). Similar findings were reported in another constipated-rat model that administrations with a natural medicine *Rhubarb* extract or its core functional component sennoside A would decrease the AQP3 expression in the colon (Kon et al., 2014). The laxative effect of *Rhubarb* extract might be associated with the major metabolite of sennoside A rheinanthrone, which could activate the macrophages to secret prostaglandin E2 as a paracine factor for decreasing AQP3 expression in colon mucosa epithelial cells (Kon et al., 2014). Moreover, sennoside A (2.6 mg/kg for 7 days) exerts strong laxative effect by regulating gut microbiota and water channels of AQP1 and AQP3 in the colon of mice (Zhang et al., 2023). These results indicated that most stimulant laxatives could decrease AQP3 expression in the colon for alleviating constipation. Nevertheless, another study indicated that high doses of sennosides displayed prominent laxative actions by downregulating most AQPs (AQP4, 5, 6, 7, and 8) expression in the colon but upregulating AQP3 expression in diarrhea-rats (Cao et al., 2018). As sennoside A are the main components of sennosides, and could be metabolized into rhein, emodin, and aloe-emodin (Sakulpanich and Gritsanapan, 2009). A previous study also showed that the laxative effect of emodin was associated with the increased expression of AQP3 in mice colon and HT-29 cells by regulating PKA/p-CREB signal pathway (Zheng et al., 2014). Based on the aforementioned studies, the variations of different results may be related to the doses of treatments, delivery method, administration period, experiment design, and managements.

Notably, increasing data has indicated important relationship between AQP3 and prostaglandin E2 during the treatment of constipation. For example, the laxative effect of bisacodyl was attributable to decreased AQP3 expression in the colon induced by increased prostaglandin E2 secretion from macrophages (Ikarashi et al., 2011b). Similarly, orally administering a purifed resin glycoside fraction from pharbitis semen to rats, could mediate its laxative effect by downregulating AQP3 expression through the cyclooxygenase-2-mediated secretion of prostaglandin E2 (Zhu et al., 2019). These data indicated that the decreased expression of AQP3 by prostaglandin E2 stimulation would inhibit water transfer from the luminal to the vascular side, thereby leading to laxative effects of the above mentioned stimulant laxatives (Ikarashi et al., 2011b; Kon et al., 2014; Zhu et al., 2019).

### 5.3 Effect of serotonergic agents for constipation on the expression of AQP3

The broadly distributed 5-HT4 receptors in the gastrointestinal tract have been well recognized as an important target for treating constipation and intestinal inflammation (Mawe et al., 2022). Serotonergic agents such as fluoxetine are usually displaying the selective laxative action on 5-HT4 receptors. Recent study has suggested that the enhancement of intestinal motility could be augmented by treatment with tryptophan-synthesizing bacteria through increased 5-HT signaling pathway with involvement of 5-HT4 receptor (Legan et al., 2023). Thus, the 5-HT4 receptor agonists used to treat functional constipation and colitis may involve the increased colonic motility by stimulation of serotonin release from enterochromaffin cells (Mawe et al., 2022) as well as the regulation of synthetic enzyme tryptophan hydroxylase in the intestine (Mawe and Hoffman, 2013). Previous study has shown that pretreatment with fluoxetine (inhibitor of 5-HT reuptake transporter) inhibited the upregulated AQP3 mRNA expression in the colon of rats with constipation (Kon et al., 2015).

Increasing evidence has suggested that probiotics and prebiotics display positive effects on colonic transit and defaecation frequency during functional constipation by targeting the VIP-cAMP-PKA-AQP3 signaling pathway (Cong et al., 2019; Tan et al., 2021). Specially, a mixture of probiotic containing *Bifidobacterium breve* DM8310, *Lactobacillus acidophilus* DM8302 and *Lactobacillus casei* DM8121 at a ratio of 1:1:1 could increase the fecal water content and ameliorate loperamide-induced constipation by regulating AQP3 expression in rat colon (Deng et al., 2018). Interestingly, postbiotic of hawthorn-porbiotic could improve intestinal movement and elevated AQP3, epithelial sodium channel gamma and mucin-2 expression in the colon for regulating intestinal water and sodium metabolism in constipated mice (Wei et al., 2023). Besides, rats receiving a diet containing 5% partially hydrolyzed guar gum (a watersoluble dietary fiber) had lower constipation symptoms probably by suppressing water transport from the luminal side to the vascular side through decreased colonic AQP3 expression (Kon et al., 2022). Furthermore, Cong et al., 2019 found that high specific volume polysaccharide, as a new type of dietary fiber, could regulate intestinal water metabolism in constipated rats by lowering the colonic AQP3 expression via cAMP-PKA pathway, and highlighted important role of the brain-gut-microbiome axis in the pathogenesis of constipation. Thus, these results indicate the potential effectiveness of probiotics and prebiotics on alleviation of constipation at least by partially targeting the colonic AQP3 expression. Notably, investigators have shown that dietary *Lactobacillus rhamnosus* GG significantly relieved gastrointestinal motility disorders by regulating 5-HT4R expression in a constipation mouse model (Gu et al., 2022). In line with this, a similar study has confirmed the involvement of 5-HT pathway and alteration of the gut microbiota in alleviating constipation by *Ligilactobacillus salivarius* Li01 (Qiu et al., 2022). Further evidence supporting this finding comes from studies in constipated-rats demonstrating the laxative effects of herbal extracts, including hot water extract of M. oleifera leaves (Li et al., 2023) and Chinese medicine Houpo Paiqi mixture (Wang J. et al., 2023), were related to gastrointestinal motility and intestinal fluid secretion via the modulation of 5-HT pathway. Another study also indicated that the yellow tea extract consumption protected against operamide-induced constipation in mice partially through the upregulation of 5-HT3 and 5-HT4 expression and downregulation of AQP3 and AQP4 expression in the colon (Cao et al., 2021). However, due to the varieties of probiotic strains, prebiotic, and complex ingredients of herbal extracts, their regulation on AQP3 expression may differ as well. Thus, further studies are required to reveal the potential mechanism of specific probiotic, prebiotic and herbal extract treatments on 5-HT4R expression through regulation of gut microbiota and and clarify their interactions with AQP3 regulation for constipation.

### 5.4 Effect of lubricating agents for constipation on the expression of AQP3

Lubricating agents (such as mineral oil, liquid paraffin, glycerol, and glycerol enema, etc.) have been commonly used to alleviate the constipation without changing the intestinal fluid homeostasis. For example, olive oil ointment can be used as a safe, well-tolerated, and effective herbal preparation in children with functional constipation (Arman-Asl et al., 2021). Moreover, the daily use of both mineral oil and plant oils (olive oil or flaxseed oil) was effective in the treatment of constipation in patients undergoing hemodialysis (Ramos et al., 2015). Currently, there have still been relatively few studies investigating the lubricating agents for constipation on the expression of AQP3. However, in one such study concerning a mouse model of constipation established by diphenoxylate, the authors found that treatment with rhein has been shown to effectively relieve the symptoms of constipation by reducing the expression of AQP3 in the colonic mucosa (Sun et al., 2018).

### 5.5 Effect of other agents for constipation on the expression of AQP3

Previous study has indicated that the total diterpenoids extracted from the roots of *Euphorbia pekinensis* could increase water content in both fecal and colonic samples followed by severe diarrhea in mice, which might be associated with the upregulation of AQP3 and AQP4 in the colon (Wang et al., 2017). Notably, Pekinenin C, as a casbane diterpenoid in *E. pekinensis*, has also been shown to induce inflammation and increase both AQP3 mRNA and protein expression in HT-29 cells alone (Wang et al., 2017) or co-culturing with RAW 264.7 cells (Yu et al., 2018). It should be noted that the laxative effects of Maren pills (Zhan et al., 2020) or Pekinenin C (Yu et al., 2018) in alleviating constipation may be associated with the increased expression of AQP3 via nuclear factor κB (NF-κB) signal pathway. Moreover, a recent study reported that the citric acid-enriched extract of ripe *Prunus mume* (Siebold) effectively improved the symptoms of constipation without causing diarrhea and tolerance by regulating the expression of AQP3 and prostaglandin E2 in the rat colons (Na et al., 2022).

Interestingly, the seasonal changes could affect the colonic expression levels of AQP3, AQP4, and AQP8 through cAMP/PKA pathway to affect the intestinal water metabolism, which resulted in seasonal differences in intestinal flora (Li et al., 2023). However, mulberry (*Morus atropurpurea*) fruit had a protective effect against diphenoxylate-induced constipation through modulation of gut microbiota and decreased expression of AQP3, AQP4, AQP8, and AQP9 in the colon of mice (Hu et al., 2019). Besides, naringenin, a natural flavonoid widely found in citrus fruits and tomatoes, has great application potential in the treatment of constipation. Yin et al. (2018) also found that naringenin treatment at 300 mg/kg significantly increased the AQP3 mRNA and protein expression levels in the colon of mice, mainly in the apical and lateral mucosal epithelial cells, which were positively associated with the increase in fecal water content and the relief of constipation. Whereas a naturally occurring flavone luteolin effectively reversed loperamide-induced upregulation of AQP3, AQP4, and AQP8 in the colon and greatly alleviated the functional constipation in mice (Wang et al., 2023). In addition to plant extracts, another study indicated that oral administration of snail-derived mucin extract ameliorated constipation symptoms induced by loperamide in rats by partially downregulating the expression of AQP3 and AQP8 (Kim et al., 2023). Notably, due to the variations of the constipation model, treatment doses, sampling and analysis strategies, the regulation of other agents for constipation on AQP3 expression may involve different mechanisms. While the exact mechanisms for these agents for constipation on the expression of AQP3 are still unclear, which requires more investigations in future studies.

## 6 Role and regulation of AQP3 in intestinal inflammation

Increasing evidence has shown that decreased AQP3 expression is associated with gastrointestinal infections and plays an important role in the inflammatory process of several human diseases (Meli et al., 2018; Sisto et al., 2019). For example, inflammatory bowel diseases often involve with dysfunctions in electrolyte and water transport in the gut. Previous study has showed that the AQP3 expression at both mRNA and protein levels was significantly reduced in ileum, proximal colon and distal colon of 2,4,6-trinitrobenzene sulphonic acid-induced rat colitis, indicating its potential roles in the pathogenesis of inflammatory bowel disease (Zhao et al., 2014). Another study also found that the AQP3 mRNA expression was reduced in the colon of rats with irritable bowel syndrome via NF-κB pathway, which might be associated with liquid water metabolic abnormalities and intestinal permeability alterations (Chao and Zhang, 2017). Furthermore, the expression of AQP3 was significantly reduced in the ileal mucosa of patients with Crohn’s disease at early stage (Ricanek al., 2015). Previous study has also confirmed that AQP3, AQP7, AQP10, and AQP11 mRNAs were significantly decreased in duodenal biopsies of untreated celiac disease patients (Laforenza et al., 2010). It was also found that the transcription and protein expression of AQP3 in HT-29 cells were decreased by lipopolysaccharide challenge in a dose- and time-dependent manner via p38/c-Jun N-terminal kinase signaling pathway (Li et al., 2015). Previous study in young pigs also showed that the AQP3 mRNA and protein expression in the jejunum was significantly decreased after lipopolysaccharide challenge, while dietary α-ketoglutarate supplementation did not significantly attenuate the jejunal expression of AQP3 in lipopolysaccharide-challenged piglets (He et al., 2017). Collectively, most of these aforementioned studies indicated that AQP3 might be involved in the pathogenesis of inflammatory bowel diseases, and its expression may decrease in response to intestinal inflammation.

Inflammatory cytokines are closely involved in intestinal inflammation with disordered ion and water transport. Tumor necrosis factor alpha (TNF-α) downregulated the constitutive transcriptional activity of the AQP3 promoter (containing the TATA-box and SP1 site), probably via the regulation of methyl ethyl ketone (MEK)/extracellular regulated protein kinases (ERK) and NF-κB signaling pathways by suppression of the specificity protein transcription factor 3 (Sp3) in HT-29 cells (Peplowski et al., 2017). Moreover, interferon gamma (IFN-γ) has been demonstrated to suppress the expression of AQP3 in the enteroids derived from human colonic biopsies as well as in HT-29 cells through increased acetylation or decreased deacetylation in AQP3 promoter (Peplowski et al., 2018). By using luciferase reporter system and chromatin immunoprecipitation analysis, they also showed that the constitutive expression of AQP3 by IFN-γ treatment in HT-29 cells was dependently regulated by Sp3, since Sp3 could bind to the AQP3 promoter (Peplowski et al., 2018). Moreover, the expression of AQP3 transcript could be inhibited by a PKA inhibitor of cAMP pathway and was upregulated by VIP in HT-29 cells (Itoh et al., 2003), which confirmed previous reports on regulating AQP3 expression through cAMP pathways as mentioned above. Interestingly, another study also indicated that AQP3 in macrophage was involved in NOD-like receptor thermal protein domain associated protein 3 (NLRP3)-inflammasome activation, and AQP3 inhibition or silencing could block the setting of inflammatory responses and decreased the production of interleukin-6 (IL-6), proIL-1β, and TNF-α (da Silva et al., 2021). These results suggested that the inflammatory mediators in the gut lumen may regulate the expression of AQP3 by interacting the AQP3 promoter regions with transcription factors such as SP3, with its complex regulatory mechanisms currently not fully understood.

Apart from the changes of AQP3 expression, previous study showed that AQP3 has one N-glycosylation site, and the misrouting of AQP3 structure may result in fluid imbalance, thus impacting Crohn’s disease and ulcerative colitis (Cohly et al., 2008). This was confirmed in another study since glycosylation was essential for cell surface expression of AQPs (Camilleri et al., 2019).

## 7 Role and regulation of AQP3 in intestinal barrier function

Increasing evidence has suggested that AQP3 plays a critical role maintaining the normal intestinal integrity and the homeostasis of intestinal epithelial barrier function. Specially, knockdown of AQP3 in the Caco-2 cells resulted in an increase in bacterial translocation and epithelial paracellular permeability accompanied with a reduction of transendothelial electrical resistance and decreased expression of tight junction proteins (e.g., claudin-1 and occludin) (Zhang et al., 2011). Another study also indicated that the expression levels of AQP3 and occludin were significantly reduced in intestinal mucosa of septic mice accompanied by an increase of plasma diamine oxidase concentration, indicating AQP3 might alleviate intestinal damage caused by sepsis (Zhu et al., 2019). Recent study has also shown that dietary berberine supplementation significantly upregulated the mRNA expression of zona occludens 1 (ZO-1) and occludin in ETEC K88-challenged piglets (Zhu et al., 2023). Moreover, daidzein could reversed lipopolysaccharide-induced downregulation of ZO-1, occludin, claudin-1, and AQP3 expression, maintained tight junction and integrity of Caco-2 cells (Zhang et al., 2022). Interestingly, microRNA-874 could promote intestinal barrier dysfunction with increased paracellular permeability and altered expression of tight junction proteins (e.g., occludin and claudin-1) by suppressing the expression of AQP3 after binding to the 3′ untranslated region (UTR) of *AQP3* mRNA as shown in Caco-2 cells (Zhi et al., 2014). Additional study from the same research group showed that human long noncoding RNA H19 may function as a competing endogenous RNA to suppress the AQP3 expression by sponging microRNA-874 in maintaining the intestinal barrier function, indicating an interaction of H19 with microRNA-874 to post-transcriptionally regulate the AQP3 protein (Su et al., 2016). In addition, the tight junction protein ZO-1 expression was decreased in AQP3 knockdown IPEC-J2 cells in response to PEDV infection (Wang et al., 2022). These updated studies indicated that AQP3 may function as a new target for improving the intestinal barrier function.

## 8 Role and regulation of AQP3 in intestinal proliferation and migration

Interestingly, the AQP3-deficient mice displayed a reduced cell proliferation of enterocytes and developed severe colitis (Thiagarajah et al., 2007), probably due to an impaired transport of glycerol for maintaining normal production of ATP energy (Hara-Chikuma and Verkman, 2008). Similarly, AQP3 deficiency leads to glycerol uptake reduction and decreased energy and lipid production, thereby impairing the proliferation in gastric cancer cells (Li et al., 2016). This was true when oral glycerol administration could effectively improve survival and reduce the severity of colitis in AQP3-null mice (Thiagarajah et al., 2007). Moreover, AQP3 facilitates cell migration, proliferation and epithelialization during wound healing in the skin of pigs (Hamed et al., 2017). Interestingly, resveratrol effectively inhibited the proliferation of normal human epidermal keratinocytes by downregulating the expression of AQP3 via the silent mating type information regulation 2 homolog 1 (SIRT1)/aryl hydrocarbon receptor nuclear translocator (ARNT)/ERK dependent pathway (Wu et al., 2014). Similarly, AQP3 could also promote the migration and invasion of human extravillous trophblast cells (Nong et al., 2021).

On the other hand, AQP3 overexpression induced by human epidermal growth factor could facilitate colorectal carcinoma cell migration, while phosphatidylinositol 3-kinase (PI3K) and protein kinase B (Akt) inhibitor would inhibit the upregulated expression of AQP3 in human colorectal carcinoma cells, indicating the important role of AQP3 in cell growth and migration by regulation of PI3K/Akt signaling pathway (Li et al., 2013). In addition, AQP3 overexpression modified the cell cycle pattern, increased the cell proliferation, and reduced cell apoptosis by regulating cell volume and complexity as well as H_2_O_2_ permeability in mammalian cells (Galan-Cobo et al., 2015). Importantly, another study also indicated that the changes in AQP3 protein expression could cause alterations in cell membrane fluidity of Caco-2 cells and thus affect drug absorption rates (Ikarashi et al., 2019b). Cell growth and migration induced by epidermal growth factor stimulation were attenuated in AQP3 knockdown cancer cells A431 and H1666 (Hara-Chikuma et al., 2016). Both *in vitro* and *in vivo* studies showed that the expression level of AQP3 in breast cancer cells was related to their migration ability (Satooka and Hara-Chikuma et al., 2016). Previous results also suggested that overexpression of AQP3 reduced cell apoptosis via the ERK1/2 pathway in nucleus pulposus cells (Zhang et al., 2022).

Notably, microRNA-874 inhibited AQP3 expression by binding to the UTR of AQP3 mRNA in gastric cancer cells, and suppressed proliferation, migration and invasion for tumorigenesis (Jiang et al., 2014). Similarly, miR-877 has been found to be negatively correlated with AQP3 mRNA expression in gastric cancer tissues, and targeting AQP3 through microRNA-877 would suppress gastric cancer development and progression (Zhu et al., 2020). However, whether AQP3 could mediate the proliferation and migration of porcine intestinal epithelial cells and how the microRNA modulates its expression remains unknown.

## 9 Role and regulation of AQP3 in intestinal oxidative stress and autophagy

### 9.1 AQP3 permeability to H_2_O_2_ and its role and regulation in intestinal oxidative stress

It is suggested that all cells could produce and consume H_2_O_2_ (Forman et al., 2016). On the one hand, H_2_O_2_ occurs in normal metabolism in mammalian cells (Sies and Chance, 1970), and functions as a central redox signaling molecule in physiological oxidative stress (Sies, 2017). The production of H_2_O_2_ can be generated from the NADPH oxidase (Nox) enzyme in the plasma membrane, the oxidative phosphorylation in mitochondrial respiratory chain, and diverse oxidases, as well as the oxidative protein folding in the endoplasmic reticulum and the peroxisomes (Kakihana et al., 2012; Sies, 2017). On the other hand, the concentration of H_2_O_2_ within cells could be more than 100-fold lower than extracellular concentrations due to the presences of catalases, peroxidases, and peroxiredoxins for the elimination of H_2_O_2_ (Sies, 2017). Usually, the intracellular physiological range of H_2_O_2_ likely spans between 1 and 10 nM for maintaining the normal proliferation, migration and angiogenenesis, while adaptive stress and oxidative stress occur at higher concentrations (10–100 nM) (Sies, 2017). However, the supraphysiological concentrations of intracellular H_2_O_2_ (>100 nM) would cause inflammation, growth arrest and cell death (Sies, 2017). Interestingly, the plasma concentration of H_2_O_2_ is about 1–5 μM range as summarized in a systemic literature review (Forman et al., 2016). Thus, the dose-dependent effects of H_2_O_2_ concentration in maintaining cellular redox signaling and oxidative stress are important to the physiology and pathophysiology of human and animal cells.

It seems that the evidence for H_2_O_2_ transport through biological membranes is controversial based on the available literature. Simple diffusion (free diffusion) was thought to be the primary approach of H_2_O_2_ transmembrane transport (Crompton, 1999). However, current evidence has indicated that H_2_O_2_ is a molecule with similar chemical properties to water (e.g., chemical composition elements, hydrogen bond stability, and polarity) and easily soluble in water, and thus could traverses cellular membranes through specific AQPs channels called peroxiporins (Lismont et al., 2019). The earlier findings should be dated to 2000 when Henzler and Steudleused the isolated internodal cells of *Chara corallina* to prove the permeation of H_2_O_2_ across a cell membrane probably mediated by water channels, since the permeability of both water and H_2_O_2_ were substantially reduced by HgCl_2_ known as the inhibitor of water channels (Henzler and Steudle, 2000). Later, Bienert et al. for the first time identified specific AQPs to facilitate the diffusion of H_2_O_2_ across membranes (Bienert et al., 2006; 2007). To date, several peroxiporins have been identified including AQP3, AQP5, AQP6, AQP7, AQP8, AQP9, and AQP11 with permeability to H_2_O_2_ (Rodrigues et al., 2019; Bestetti et al., 2020; Wragg et al., 2020; Krüger et al., 2021; Pellavio et al., 2022; Ma et al., 2023), which may play important roles in oxidative stress regulation. Notably, the driving force of H_2_O_2_ permeability across membrane mediated by AQPs may be related to the gradients between extracellular and intracellular H_2_O_2_ concentrations as indicated above (Sies, 2017).

Importantly, human AQP3 facilitated H_2_O_2_ transport through its NPA motif was confirmed using a combination of metadynamics and transition path sampling methods (Wragg et al., 2020). Considering this role of NPA motifs, it could be explained why AQP3 mediated route of H_2_O_2_ transport could be more efficient than simple diffusion. Until now, numerous studies have proved the important function of AQP3 as one of the important peroxiporins to mediate the transport of H_2_O_2_ in various cells. For example, previous study has demonstrated that AQP3-mediated cellular H_2_O_2_ uptake was essential for TNF-α-induced NF-kB activation, while AQP3 deficiency would suppresses NF-kB activation and H_2_O_2_ induction in epidermal keratinocytes (Hara-Chikuma et al., 2015). Moreover, T cell migration toward chemokines was highly dependent on AQP3-mediated H_2_O_2_ uptake, and might play an important role in the immune responses (Hara-Chikuma et al., 2012). Notably, AQP3-mediated H_2_O_2_ transport into fibroblasts required activation of TGF-β1, while silencing AQP3 would decrease H_2_O_2_ levels and TGF-β1 expression, and alleviated fibrosis in mice (Luo et al., 2016). Another study also revealed the oxygen-dependent regulation of AQP3 expression could be significantly enhanced by hypoxia in L929 fibrosarcoma cells (Hoogewijs et al., 2016). Furthermore, the transport of H_2_O_2_ into the endosomal lumen mediated by AQP3 can regulate the endosomal lipid peroxidation closely related to oxidative stress (Nalle et al., 2020).

Increasing data has suggested that AQP3 may mediate the transport of H_2_O_2_ produced by cell-surface Nox enzyme. For example, Miller et al. (2010) used the genetically encoded fluorescent sensor to detect H_2_O_2_, and confirmed that AQP3 could mediate Nox-derived H_2_O_2_ uptake upon growth factor stimulation to regulate downstream intracellular signaling. Notably, another study also indicated that AQP3 could associate epidermal growth factor receptor and Nox2 to increase cellular H_2_O_2_ uptake (Hara-Chikuma et al., 2016). Consistently, the extracellular H_2_O_2_ produced by Nox2 activation could be transported into breast cancer cells via AQP3 (Satooka and Hara-Chikuma et al., 2016). Another study also reported an upregulation of Nox1/2 and downregulation of AQP3 under oxidative stress induced by H_2_O_2_ (Dong et al., 2020). These results indicated that Nox1/2 is an important source of H_2_O_2_, and plays important roles in AQP3-mediated H_2_O_2_ permeability.

Intestinal oxidative stress often occurs with inflammation and disturbs intestinal homeostasis. In diabetic mice, the increased oxidative stress was found to be accompanied with decreased AQP3 expression (Ikarashi et al., 2019a). Previous study has demonstrated that oxidative stress insult modulated the expression of peroxiporins which were associated with oxidative stress resistance in colon cancer cell lines (Cipak Gasparovic et al., 2021). These authors found that under oxidative stress exposure, the AQP3 expression in HCT 116 cells was significantly increased after treatment with 100 μM H_2_O_2_ compared to other colon cancer cell lines (Caco-2, HT-29, and SW620) (Cipak Gasparovic et al., 2021). This might be due to the fact that AQP3 could function as peroxiporin to mediate the entry of extracellular H_2_O_2_ into colonic epithelial cells, since defective repair after wounding and impaired H_2_O_2_ responses to pathogen infection were observed in both AQP3-deleted Caco-2 cells and AQP3 knockout mice (Thiagarajah et al., 2017). Indeed, the H_2_O_2_ entry through AQP3 would regulate extracellular Ca^2+^ influx during the honey-mediated wound healing (Martinotti et al., 2019). This was in accordance with another study that oxidative damage could significantly decrease the AQP3 expression and increase apoptosis of nucleus pulposus cells, while AQP3 over-expression could partially attenuate the cellular apoptosis through p38 mitogen-activated protein kinase (MAPK) pathway under oxidative damage (Xu et al., 2018). Collectively, these data indicated that AQP3 could function as an important regulator of redox status in intestinal epithelial cells, and may represent an promising targeting for the development of potential managements for alleviating the intestinal oxidative-associated disorders or diseases in human and animals. It should be noted that diverse cellular stresses reversibly inhibited the AQP8-dependent transport of H_2_O_2_ and water to impact cell growth and survival (Medraño-Fernandez et al., 2016). However, due to the concern of H_2_O_2_ as a highly reactive molecule, more functional researches are needed to clarify whether H_2_O_2_ could transports via AQP3 without oxidizing lipids and proteins and the water channel itself, as well as the regulation mechanism of H_2_O_2_ production and elimination in intestinal epithelial cells.

Previous study has shown that the protective effect of AQP3 in alleviating the oxidative damage might involve the elimination of intracellular reactive oxygen species (ROS) (Laforenza et al., 2016). Many studies on plant extracts have confirmed the involvement of AQP3 in regulating the oxidative stress. For example, pterostilbene, as a dimethylated analog of resveratrol, could significantly reduce particulate matter-induced intracellular ROS production and attenuate oxidative stress, and increase AQP3 expression in keratinocytes (Teng et al., 2021). Interestingly, monomethylfumarate treatment attenuated the generation of ROS following H_2_O_2_ treatment, and stimulated the increased AQP3 mRNA and protein expression in keratinocytes (Helwa et al., 2017). Similarly, Pellavio et al. (2017) has reported several natural antioxidant compounds reduced the H_2_O_2_ content of heat-stressed cells or prevent H_2_O_2_ cellular entry by regulation the functional properties of AQPs (1, 3, 8, and 11). The evidence of AQP3 in regulation of intestinal oxidative has also been recognized. By using an *in vitro* CELLigence system and *in vivo* evaluation in a colitis model of mice, Dong et al. (2020) found that compared to other plant bioactive components, quercetin most effectively alleviated the decreased cell index, reversed cell damage, and decreased reactive oxygen species and apoptosis ratio caused by H_2_O_2_. They concluded that the protective effects of quercetin in intestinal oxidative damage might be related to modulation of glutathione and H_2_O_2_ production and transport via Nox1/2 and AQP3, respectively (Dong et al., 2020). Importantly, meyloperoxidase (MPO) enzyme could use H_2_O_2_ to oxidize chloride for catalyzing the production of a strong oxidant hypochlorous acid (Dhiman et al., 2009). A recent study has indicated that quercetin effectively attenuated human vascular endothelial dysfunction by inhibiting MPO/H_2_O_2_-mediated hypochlorous acid production as well as Nox function (Li et al., 2023). Thus, the application of natural compounds may be helpful for reducing brain ischemia injury by targeting MPO-mediated oxidative stress and inflammation (Chen et al., 2020).

However, there were few evidence reporting the potential involvement of other antioxidant enzymes in regulating intestinal oxidative stress by targeting AQP3. Nonetheless, it should be noted that glutathione was supposed as a key determinant in the elimination of peroxides by the intestine (Aw, 2005). A previous study has demonstrated that *Centella asiatica* extracts were able to increase the content of glutathione and superoxide dismutase while reducing intracellular ROS generation, accompanied with downregulation of inflammatory factors and upregulation of AQP3 expression (Lin et al., 2023). Importantly, ulcerative colitis is often associated with oxidative stress and inflammation. For the therapies of ulcerative colitis, synbiotics could inhibit the oxidative stress’ effects and alleviate the colonic inflammation by lowering the ROS and improving the level of antioxidant enzymes such as catalase, glutathione peroxidase, and superoxide dismutase (Ashique et al., 2023). Moreover, a previous study found that the strong antioxidant properties of cell-free culture supernatant from the *Bifidobacterium infantis* strain on the skin, might be associated with reduced intracellular ROS and malondialdehyde contents and improved activities of catalase, glutathione peroxidase, and superoxide dismutase as well as upregulation of AQP3 in H_2_O_2_ -treated HaCaT cells after treatment (Ma et al., 2022).

### 9.2 Role and regulation of AQP3 in intestinal autophagy

Autophagy is closely associated with the development of intestinal oxidative stress, and may involve with the change of AQP3 expression. Since AQP3 may also mediate arsenic uptake, and upregulation of AQP3 expression would contribute to arsenic-induced autophagy in keratinocytes (Yu et al., 2021). The previous *in vitro* study has also found that AQP3 overexpression promoted the conversion of autophagy-related protein LC3-I to LC3-II and upregulated the ATG5 and Beclin-1 expression in gastric cancer cell line AGS, whereas AQP3 knockdown inhibited the transformation of LC3-I to LC3-II in MGC803 and SGC7901 cells, indicating that AQP3 expression regulated autophagy differently in different gastric cancer cells (Dong et al., 2016). Interestingly, AQP3 silencing inhibited cell viability and autophagy in gastric cancer cells while AQP3 overexpression activated autophagy via Krüpper like factor 5 as confirmed by chromatin immunoprecipitation and dual luciferase reporter assays (Dai et al., 2023). Moreover, increasing data has indicated that ETEC infection caused significant autophagy in porcine intestinal epithelial cells (Tang et al., 2014; Xia et al., 2019; Luo et al., 2022). In addition to ETEC, a recent study showed that PEDV induced autophagy in Vero CCL81 cells (Ren et al., 2022). As mentioned above, whether ETEC or PEDV-induced autophagy is associated with regulation of AQP3 in the gut of piglets remained unclear and needs further investigations.

Previous study has shown that the diffusion and uptake of exogenous H_2_O_2_ mediated by AQP3 significantly increased the intracellular reactive oxygen species levels and inhibited rapamycin-induced autophagy via activating the Akt/mammalian target of rapamycin (mTOR) pathway in lung adenocarcinoma cells (Wang et al., 2021). Indeed, another study has also confirmed that mTOR signaling pathway could regulate AQP3 expression, and overexpression of AQP3 could induce autophagy and promote proliferation and migration of gastric cancer cells (Yu et al., 2022). Moreover, latent membrane protein 2A (LMP2A) induced the phosphorylation of ERK, which thereby activated DNMT3a transcription and induced loss of AQP3 expression through CpG island methylation of AQP3 promoter in Epstein-Barr virus-associated gastric carcinoma (Wang et al., 2019). This was true since LMP2A could downregulate AQP3 by inhibiting mTOR signaling pathway to promote autophagy of gastric cancer cells (Yu et al., 2022). These studies suggested that LMP2A may regulate the AQP3 expression and activate autophagy by targeting mTOR pathways. However, it remained unclear how mTOR pathway might regulate autophagy of intestinal epithelial cells in human and animals by targeting AQP3, and therefore needs further investigations.

## 10 Future aspect and concluding remarks

**FIGURE 2:**
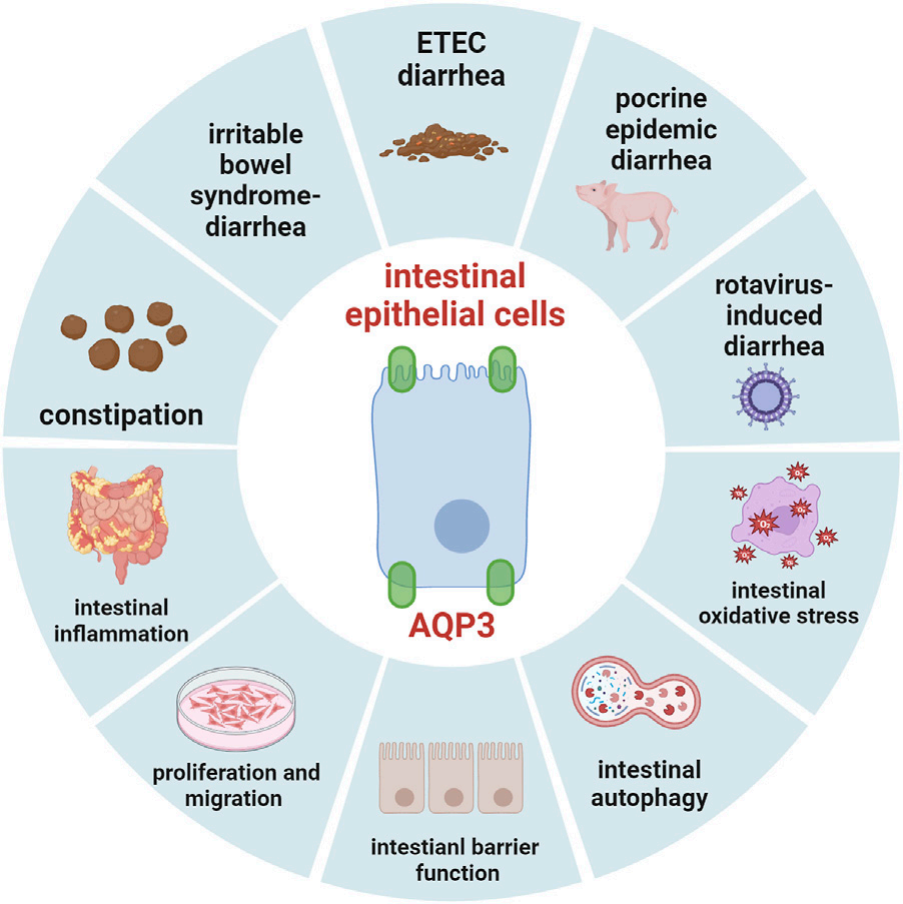
The summary of role of AQP3 in maintaining gut health.

Current progress has indicated that AQP3 may act an important target for the disorders that involve disruption of intestinal fluid homeostasis (as summarized in Figure 2, created with BioRender.com), such as diarrhea, constipation, inflammatory bowel disease, and irritable bowel syndrome. It has been recognized that AQP3 is essential for maintaining intestinal proliferation and migration as well as intestinal barrier function, and mediates the transport of H_2_O_2_ via Nox, which thereby plays important roles in intestinal oxidative stress and autophagy. Great progress has been made through dietary nutrients to regulate intestinal health by targeting AQP3. However, significant work should be done concerning the specific roles of AQP3 mediating the potential protective intervention strategies for regulating gut health in human and animals under physiological and pathological conditions. Importantly, histone methylations and acetylations or modifications of DNA methylation status may affect gene expression of AQP3. Thus, given the aformentioned findings and important implications, more investigations are necessary to elucidate the potential mechanism of AQP3 in gut health at both post-transcription and post-translation levels in future studies.
